# The Characteristics and Genome Analysis of vB_AviM_AVP, the First Phage Infecting *Aerococcus viridans*

**DOI:** 10.3390/v11020104

**Published:** 2019-01-26

**Authors:** Hengyu Xi, Jiaxin Dai, Yigang Tong, Mengjun Cheng, Feiyang Zhao, Hang Fan, Xinwei Li, Ruopeng Cai, Yalu Ji, Changjiang Sun, Xin Feng, Liancheng Lei, Sadeeq ur Rahman, Wenyu Han, Jingmin Gu

**Affiliations:** 1 Key Laboratory of Zoonosis Research, Ministry of Education, College of Veterinary Medicine, Jilin University, Changchun 130062, China; m15543135579@163.com (H.X.); 15204525855@163.com (J.D.); mengjun_c@163.com (M.C.); vetboss@163.com (X.L.); saycall850@126.com (R.C.); jiyalu120@163.com (Y.J.); schangjiang@126.com (C.S.); xinple@163.com (X.F.); leiliancheng@163.com (L.L.); 2 State Key Laboratory of Pathogen and Biosecurity, Beijing Institute of Microbiology and Epidemiology, Beijing 100071, China; tong.yigang@gmail.com (Y.T.); yangfan10159532@163.com (F.Z.); Fanhang11@gmail.com (H.F.); 3 College of Veterinary Sciences & Animal Husbandry, Abdul Wali Khan University, Mardan 23200, Pakistan; sadeeq@awkum.edu.pk; 4 Jiangsu Co-Innovation Center for the Prevention and Control of Important Animal Infectious Disease and Zoonose, Yangzhou University, Yangzhou 225009, China

**Keywords:** *Aerococcus viridans*, opportunistic pathogen, phage, genome analysis

## Abstract

*Aerococcus viridans* is an opportunistic pathogen that is clinically associated with various human and animal diseases. In this study, the first identified *A. viridans* phage, vB_AviM_AVP (abbreviated as AVP), was isolated and studied. AVP belongs to the family Myoviridae. AVP harbors a double-stranded DNA genome with a length of 133,806 bp and a G + C content of 34.51%. The genome sequence of AVP showed low similarity (<1% identity) to those of other phages, bacteria, or other organisms in the database. Among 165 predicted open reading frames (ORFs), there were only 69 gene products exhibiting similarity (≤65% identity) to proteins of known functions in the database. In addition, the other 36 gene products did not match any viral or prokaryotic sequences in any publicly available database. On the basis of the putative functions of the ORFs, the genome of AVP was divided into three modules: nucleotide metabolism and replication, structural components, and lysis. A phylogenetic analysis of the terminase large subunits and capsid proteins indicated that AVP represents a novel branch of phages. The observed characteristics of AVP indicate that it represents a new class of phages.

## 1. Introduction

*Aerococcus viridans* is an environmental Gram-positive saprophytic bacterium that belongs to the Streptococcaceae family [1]. *A. viridans* resembles streptococci microscopically and often causes coinfections with streptococci [2,3]. Because it is often mistaken for staphylococci or streptococci, the pathogenicity of this bacterium has been underestimated [4]. In recent years, *A. viridans* has been observed to be clinically associated with different human and animal diseases [1]. *A. viridans* has been reported to cause infections in lobsters [5] and tilapia [6], and is associated with swine arthritis [7], swine meningitis [7], swine pneumonia [7], and bovine mastitis [8,9,10,11]. In humans, *A. viridans* is described as an opportunistic pathogen that can cause urinary tract infections [12], bacteremia [13], meningitis [14], septic arthritis [15], and especially endocarditis [16]. *A. viridans* has been reported to be increasingly resistant to most common antibiotics [13,17] and, importantly, vancomycin-resistant isolates have recently been described [18].

Bacteriophages (phages) are bacterial viruses that specifically recognize, infect, and replicate inside host bacteria, with more than 10^31^ phage particles reported to exist in the biosphere [19]. This massive phage diversity has a marked effect on the environment, ecology, and bacterial evolution [20]. Owing to their specific bactericidal abilities, phages have been considered as therapeutic agents since the early 1920s [21]. However, the development of this therapy has lagged due to the wide availability of antibiotics [22]. In recent years, owing to the global emergence of multidrug-resistant bacteria, the study of phage therapy has experienced a renaissance.

Although bacteriophages of most described bacteria have previously been reported, including those for *Escherichia coli* [23,24], *Listeria* [25,26], *Staphylococcus aureus* [27,28], and *Streptococcus* [29,30], among others, no *A. viridans* bacteriophage has been described.

In this study, the first bacteriophage that specifically infects *A. viridans,* named vB_AviM_AVP (abbreviated as AVP), was isolated and characterized. A genome analysis shows that AVP has extremely low similarity to other phages, bacteria, and other organisms in the database.

## 2. Materials and Methods

### 2.1. Bacterial Strains and Growth Conditions

*A. viridans* AV-X1 was isolated from clinical specimens of meningitis-infected pigs and initially cultured on TSA (trypticase soy agar, Sigma, Shanghai, China) containing 5% defibrinated sheep blood. The isolated strain was identified by 43 different biochemical tests (Table S1) (VITEK 2 Compact, France Bio, Mérieux, France) and was further confirmed as *A. viridans* by amplifying a 540-bp fragment of the 16S rRNA gene with the specific primer pair F (5′-GTGCTTGCACTTCTGACGTTAGC-3′) and R (5′-TGAGCCGTGGGCTTTCACAT-3′) [31]. The PCR product was sequenced and the resulting sequence was compared against the GenBank accession database using BLAST. The strain was stored in 30% glycerol at −80 °C and was routinely grown at 37 °C using a brain–heart infusion (BHI, Becton-Dickinson, Franklin Lakes, NJ, USA) medium.

### 2.2. Isolation of the Aerococcus viridans Phage

The isolated *A. viridans* strain AV-X1 was used as a host strain to isolate the phage from sewage samples. Sewage samples were collected from a sewer system (Changchun, Jilin, China) and were filtered with four-layer gauze. AV-X1 was cultured to the logarithmic phase in BHI broth until the OD_600 nm_ reached 0.6, after which AV-X1 and the prepared sewage samples were cocultured overnight in BHI broth at 37 °C. Subsequently, the coculture was centrifuged at 10,000× *g* for 10 min at 4 °C, and the supernatant was filtered using a sterile 0.22 µm pore-size filter (Millex-GP Filter Unit; Millipore, Bedford, MA, USA; LOT R6MA05262). To detect and purify the phage from the supernatant, a double-layer agar plate assay was carried out [32]. Briefly, a single spot was picked from the double-layer plate and added to 200 μL of the host strain grown to the log phase for amplification in 5 mL of BHI medium, and the double-layer plate method was carried out after 5 h. This was repeated three times to obtain the purified AVP phage lysate. The concentration of agar in the overlays was 0.7%. The purified phage was amplified and stored at 4 °C and at −80 °C in glycerol (3:1, *v*/*v*).

### 2.3. CsCl Density Gradient Centrifugation

Following large-scale culturing, AVP was precipitated using 10% (*w*/*v*) polyethylene glycol 8000 and 1 M NaCl. Next, the phage sample was placed at the top of a discontinuous CsCl gradient (1.32, 1.45, 1.50, and 1.70 g/mL) and centrifuged at 35,000× *g* for 3 h at 4 °C. The phage band was collected and dialyzed with a suspension medium (SM) buffer (0.01% gelatin, 100 mM/L NaCl, 50 mmol/L tris-HCl, and 10 mM/L MgSO_4_) at 4 °C.

### 2.4. TEM of vB_AviM_AVP

Purified AVP was applied to 200 mesh copper grids and negatively stained with phosphotungstic acid (2%, *w*/*v*). The morphology of AVP was examined by transmission electron microscopy (JEOL JEM-1200EXII; Japan Electronics and Optics Laboratory, Tokyo, Japan) at an accelerating voltage of 80 kV.

### 2.5. One-Step Growth Curve

After the addition of 100 μL AVP solution (3 × 10^7^ PFU/mL) into 1 mL AV-X1 culture 3 × 10^7^ CFU/mL at an multiplicity of infection (MOI) of 0.1, the mixture was incubated for 5 min at 37 °C. Next, the mixture was centrifuged at 10,000× *g* for 5 min at 4 °C. The pellet was suspended in 10 mL of BHI broth and incubated at 37 °C with shaking at 180 rpm, with samples collected every 5 min for the first 30 min, and then every 10 min until 60 min to test the concentration of the phage. The first set of samples was immediately diluted and titrated using a double-agar method. The second set of samples was processed with 1% (*v*/*v*) chloroform for 30 min prior to phage titration to release intracellular phages to determine the eclipse phase [33]. The burst size was defined as the ratio of the total number of phages liberated at the end of one cycle of growth to the number of infected bacteria [34]. This experiment was repeated in triplicate.

### 2.6. Sequencing and Bioinformatics Analysis of the AVP Genome

After the large-scale amplification of AVP, the cellular material was removed by centrifugation at 10,000× *g* for 20 min at 4 °C. Next, DNase I and RNase A were added to the supernatant at a final concentration of 1 μg/mL followed by incubation for 30 min. The supernatant was collected by centrifugation at 10,000× *g* for 20 min at 4 °C. AVP was precipitated overnight using 10% (*w*/*v*) polyethylene glycol 8000 and 1 M NaCl. The precipitated phage was resuspended in an SM buffer. Finally, an equal volume of chloroform was added for phage extraction, with the concentrated phage obtained after centrifugation (4000× *g* for 15 min at 4 °C). The genome of AVP was extracted using a viral genome extraction kit (Omega Bio-Tek Inc., Doraville, GA, USA) as per the instructions of the manufacturer. Whole-genome sequencing was performed at the Beijing Genomics Institute using IlluminaHiSeq 2500 sequencing. Newbler v.2.8 was used to assemble sequences, and the AVP genome was visualized using CLC Genomics Workbench software (CLCbio). Genes were initially predicted using the Rapid Annotation using Subsystem Technology (RAST) annotation server [35]. All predicted ORFs were manually verified by performing searches against the National Center for Biotechnology Information (NCBI) database using PSI-BLAST (the cutoff was 0.0001) and HMMER (HMMER web server: 2018 update) with a minimum *E*-value of 10^−4^ [36]. A tRNA scanner (http://lowelab.ucsc.edu/tRNAscan-SE/index.html) was used to analyze tRNAs [37]. The sequence alignment was generated using CLC Main Workbench, version 7.7.3 (CLC Bio-Qiagen, Aarhus, Denmark). The amino acid sequences of terminase large subunits and capsid proteins were aligned using PSI-BLAST with a minimum *E*-value of 10^−4^. The output was sorted according to the hit score, from which different phages having homology were selected and their nucleic acid sequences were obtained. The sequences were then classified according to the NCBI taxonomy database. The gene sequences of the terminase large subunits and capsid proteins were aligned with reference gene sequences using the ClustalW program within the BioEdit 7.0.4.1 package. Once aligned, the maximum likelihood (ML) phylogenetic trees were constructed using their nucleic acid sequences with 100 bootstrap replicates [38].

## 3. Results and Discussion

### 3.1. The Characteristics of Aerococcus viridans

The strain AV-X1, which was isolated from brain samples, formed small (≤1 mm diameter), circular, and translucent colonies with smooth surfaces with an off-white color and exhibited typical α-hemolysis on blood agar plates, showing its role as a pathogen (Figure S1a). Microscopically, AV-X1 appears as groups of four Gram-positive cocci that are occasionally paired or clustered. Further identification via biochemical tests (Table S1) indicated that AV-X1 is most likely *A. viridans*, with a 97% confidence value. AV-X1 was ultimately confirmed as an *A. viridans* strain by 16S rRNA gene sequencing (Figure S2).

### 3.2. The Biological Characteristics of vB_AviM_AVP

**Figure 1:**
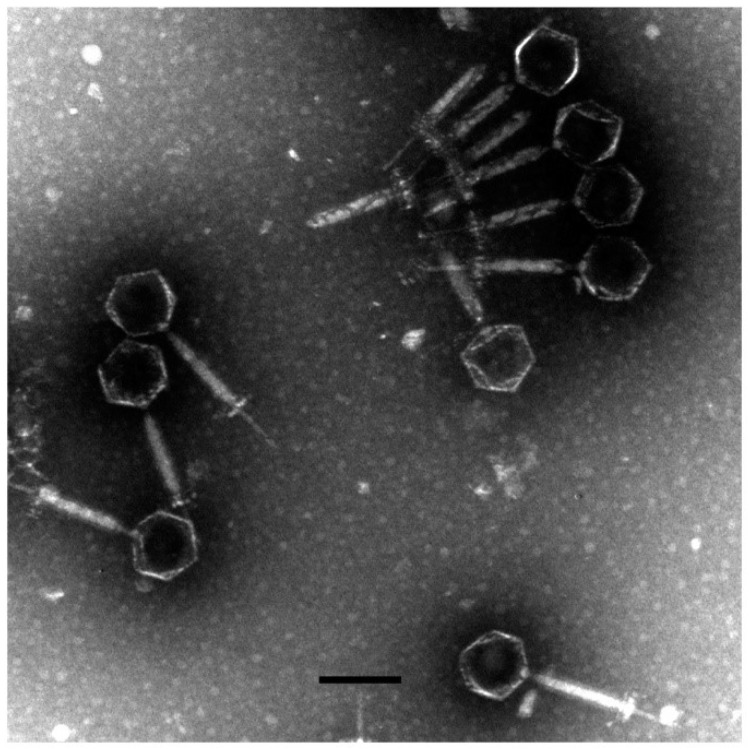
The morphology of vB_AviM_AVP (AVP). AVP was negatively stained with 2% phosphotungstic acid (PTA) and examined by transmission electron microscopy (TEM) at an accelerating voltage of 80 kV. The scale bar represents 100 nm.

**Figure 2:**
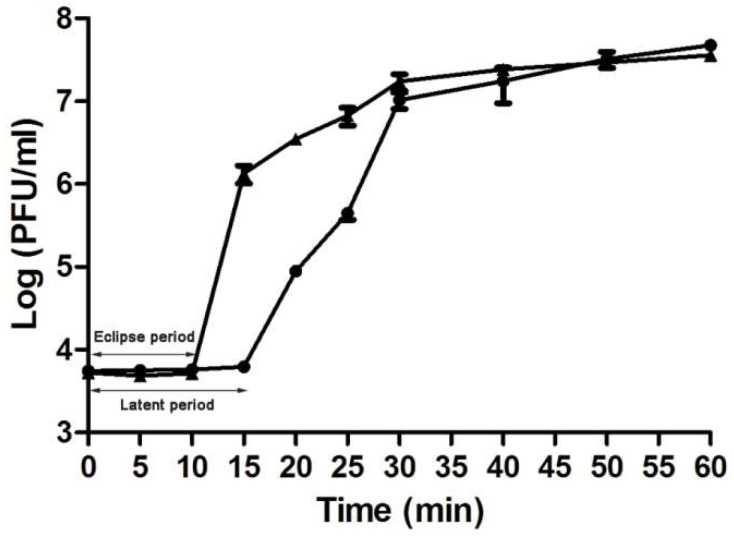
One-step growth curve for vB_AviM_AVP (AVP) in *Aerococcus viridans* AV-X1. Shown are samples treated with (▲) and without (●) chloroform at different time points. The values indicate means and standard deviations (SD) (*n* = 3).

A novel phage, named as vB_AviM_AVP (abbreviated as AVP), was isolated from sewage using AV-X1 as the host strain. AVP formed clear plaques on BHI plates after coculturing with a bacterial lawn of AV-X1 (Figure S1b). Electron micrograph images of AVP indicated that it possesses isometric, icosahedral heads and contractile tails, suggesting that AVP is a member of the Myoviridae family, as shown in Figure 1. The diameter of the AVP head is approximately 94 ± 5 nm (*n* = 3), and the tail length is approximately 189 ± 5 nm (*n* = 3). A one-step growth curve of AVP infecting AV-X1 revealed an eclipse period of 10 min, a latent phase of 15 min, and a burst size of about 139 PFU per infected cell ((4.18 × 10^7^ PFU/mL × 10 mL)/(3 × 10^7^ PFU/mL × 100 μL)), indicating that AVP is an efficient phage, as shown in Figure 2.

### 3.3. The General Characteristics of the AVP Genome

**Figure 3:**
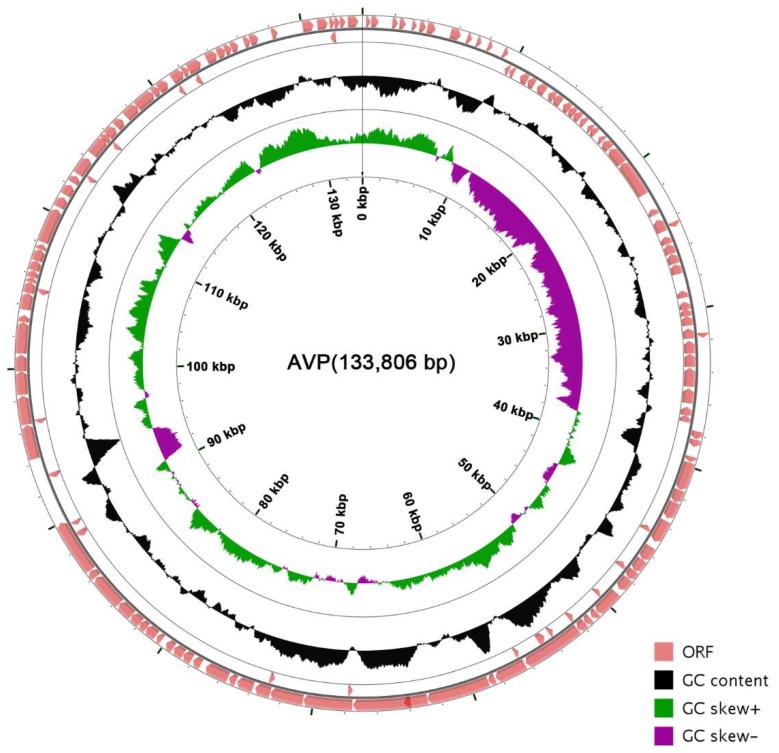
Genetic and physical organization of the vB_AviM_AVP (AVP) genome. The 165 ORFs of AVP are depicted, and the direction of transcription is indicated by arrows. The G + C content and skew of AVP are also shown. The circle map of the AVP genome was made using CGView (http://wishart.biology.ualberta.ca/cgview/) [40].

The coverage of the genome is 100% and the average depth of sequencing is 3595.36. The genome of AVP is a contiguous, 133,806-bp, double-stranded DNA sequence. The genome sequence of AVP showed low similarity (<1% identity) to those of other phages, bacteria, or other organisms in the database. The total G + C content of AVP was determined to be 34.51%, which is significantly lower than that of its host, *A. viridans* (about 39.4%) [39]. Five transfer-RNA-encoding genes were found in the genome (Table S2). A total of 165 putative ORFs were predicted in the complete AVP genome (Table S3), with ATG (135/165), TTG (24/165), and GTG (6/165) serving as start codons and TAA (100/165), TAG (58/165), and TGA (7/165) serving as stop codons. The genome order was set according to one of the determined termini. The open reading frames have a coding density of 86.45%, covering a total of 115,681 bp, and the majority of the ORFs (114/165) were observed to be transcribed on the positive strand. The arrangement of the entire genome is shown in Figure 3.

### 3.4. The Putative ORFs of AVP

**Figure 4:**
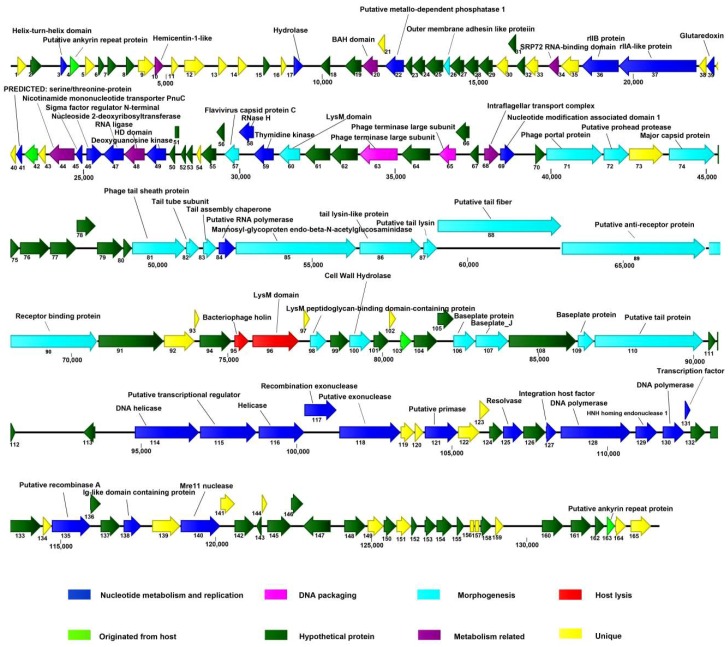
Graphical representation of the vB_AviM_AVP (AVP) genome. The 165 ORFs are depicted and the direction of transcription is indicated by arrows. Proposed modules are based on hypothetical functions predicted through bioinformatic analysis. The genome map was drawn using CLC Main Workbench, version 7.7.3 (CLC Bio-Qiagen, Aarhus, Denmark).

The putative ORFs in the AVP genome were mapped (Figure 4). Among the predicted ORFs, 69 gene products exhibiting an identity similar (≤65%) to proteins of known functions were tentatively assigned corresponding functions. In addition, 36 gene products did not match any viral or prokaryotic sequences in any publicly available database. On the basis of the putative functions of the ORFs, the genome of AVP was divided into three modules: nucleotide metabolism and replication, structural components, and lysis.

### 3.5. The Lysis Module

Three ORFs (ORF60, -96, and -98) in the AVP genome encode LysM peptidoglycan-binding domain-containing proteins, although only ORF96 is structurally adjacent to a putative holin (ORF95). Additionally, ORF100 encodes a cell wall hydrolase that hydrolyzes peptidoglycans, causing bacterial lysis. Considering that many genes encoding phage endolysins are adjacent to holin-encoding genes [28,29,30], especially phages of Gram-positive bacteria, ORF96 likely encodes an AVP endolysin. Interestingly, the lysis module (ORF95–ORF96) is located in the structure module. In addition, ORF98 encodes a protein that is homologous to the CHAP protein of *Aerococcus* sp., which may have a catalytic activity for peptidoglycan. Studies performed over the past decades have demonstrated that the LysM motif is involved in binding to bacterial peptidoglycan (PG), and LysM-containing hydrolases can hydrolyze bacterial PG [41,42]. Consequently, these putative LysM peptidoglycan-binding domain-containing proteins in AVP may work together to more efficiently hydrolyze the bacterial cell wall. The identity similarity among these four LysMs is very low, suggesting that they may act on different chemical bond of peptidoglycan. Additionally, the lack of similarity may be because no phage infecting *A. viridans* has been isolated before.

### 3.6. The Nucleotide Metabolism and Replication Module

The nucleotide metabolism and replication module of AVP is primarily located in two regions (1594–31,006 bp and 94,425–119,746 bp). Comparative analysis revealed that the following ORFs related to DNA replication were distributed in this module: DNA helicase (ORF114 and ORF116), putative transcriptional regulator (ORF115 and ORF131), exonuclease (ORF117 and ORF118), primase (ORF121), resolvase (ORF125), putative integration host factor (ORF127), polymerase (ORF128 and ORF130), recombinase A (ORF135), and Mre11 nuclease (ORF140). With respect to the 1594–31,006 bp region of this module, the putative products (ORF3, -17, -22, -36, -37, -39, -41, -45, -46, -47, -49, -58, and -59) are also possibly involved in DNA metabolism and replication.

ORF121 exhibited ~50% amino acid identity with the putative primase of the *Enterococcus* phage EFP01. Owing to the semi-discontinuous nature of DNA replication, primases are required during the initiation and throughout the process of lagging strand replication [43]. Furthermore, primase can bind to the N-terminus of helicase to form a replicator, which has a wider role in DNA replication, repair, and possibly transcription.

A putative integration host factor encoded by ORF127 was also identified in this module, which can promote the assembly of a terminase motor complex [44], act as an activator of phage DNA replication [45], and is involved in the “decision” of a bacteriophage to end up with either the lytic or lysogenic cycle [46].

In addition, PSI-BLAST analysis showed that ORF128 and ORF130 of AVP encode two putative components which show homology with the N-terminal and C-terminal parts of the whole DNA polymerase, respectively. The data of Table S3 clearly indicate that the protein encoded by ORF129 showed similarity with HNH homing endonuclease (accession no. AUO79433.1), a site-specific DNA endonuclease that promotes the horizontal transfer of a gene and its flanking DNA [47]. Introns, including the homing endonuclease-like genes of the HNH superfamily, have been observed in the terminase large subunit gene of the *Staphylococcus* phage Twort [48,49]. Thus, the gene fragment between the genes ORF128 and ORF130 may be an inserted gene fragment (Figure S3). Group I introns have been reported to be capable of encoding homing endonucleases, which are responsible for placing the intron in the correct sequence context for efficient splicing [50]. Thus, the putative protein encoded by ORF129 may be responsible for intron mobility. Very interestingly, the N-terminal (ORF128) and C-terminal (ORF130) parts of the whole DNA polymerase can serve a normal function in the normal replication of AVP.

Of note, AVP was also observed to carry a gene (ORF45) encoding RNA polymerase sigma factor N-terminal, an 8.8-kDa protein. RNA polymerase can be modified by this σ factor to recognize phage-promoter regions, promoting the expression of phage genes rather than host genes [51]. Additionally, ORF115 and ORF131 encode putative transcriptional regulators that modify RNA polymerase to recognize phage-promoter regions, regulating gene expression such that phage genes are expressed rather than host genes, similar to the sigma factor of the staphylococcal phage [51]. Noticeably, ORF140 of AVP showed similarity to the Mre11 nuclease of the *Staphylococcus* phage phiIPLA-C1C. Interestingly, Mre11 was reported to play a role in DNA damage repair [52]. The protein encoded by ORF84 shows 36% amino acid identity similarity to the putative RNA polymerase of the *Enterococcus* phage EFLK1 [53]. Interestingly, this ORF is located in the structural module.

### 3.7. The Structure and Packaging Module

The structural and packaging gene clusters were also identified in the AVP genome with the following order: terminase large subunit, terminase small subunit, portal protein, putative prohead protease, major capsid protein, and major tail sheath protein.

In particular, both the proteins encoded by ORF63 and ORF65 have short patches of similarity to other terminase large subunits. However, considering that the large subunit typically consists of 400–750 aa, while the small subunit typically contains 150–200 aa, it is thought that the 405-aa protein encoded by ORF63 is a terminase large subunit, whereas the protein product of ORF65, which only contains 181 aa, is a terminase small subunit. Of these structure-associated products, ORF71 encodes the portal protein, which can form a channel in capsids and contributes to the injection of the bacteriophage genome into the host cell [54]. The proteins encoded by ORF72 and ORF74 showed similarities to the prohead protease of the *Listeria* phage LMTA-34 and the major capsid protein of the phage phiIPLA-C1C [55], respectively. The formation of the procapsid structure (also referred to as the prohead) initiates the morphogenesis of phages. During capsid maturation, the protease contained in the prohead is activated such that the inner core is destroyed and space for the genome is liberated [56,57].

**Figure 5:**
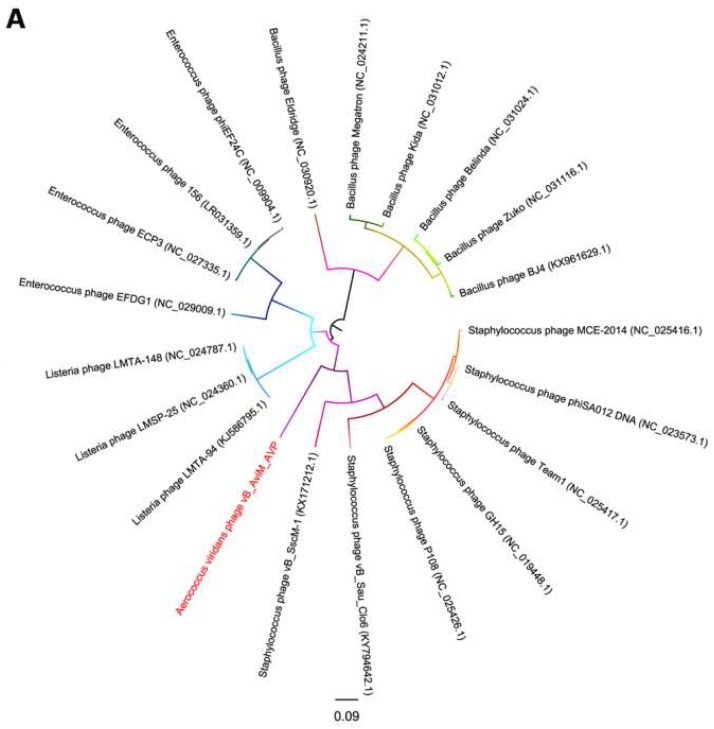

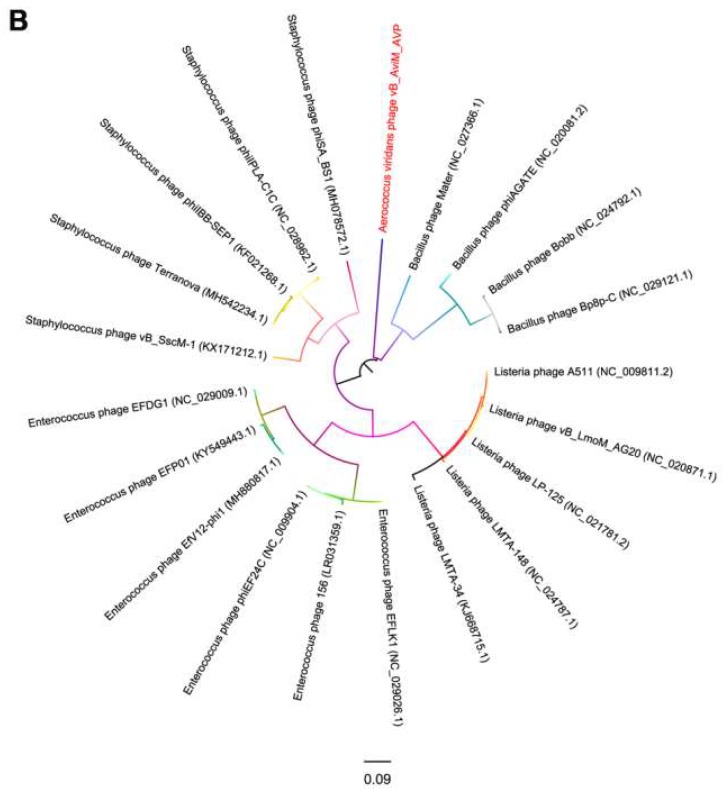
Phylogenetic tree based on the terminase large subunits (**A**) and capsid proteins (**B**) of selected phages. Both the terminase large subunits and capsid proteins were compared using PhyML version 3.0, and the phylogenetic trees were generated using the maximum likelihood method with 100 bootstrap replicates.

Due to the conservation of the terminase large subunits and capsid proteins, they were used to generate a phylogenetic tree [58,59]. Given the small datasets, *Bacillus* phages are selected as the outgroup and we used maximum likelihood (ML) to generate the phylogenetic tree. The phages used in the phylogenetic tree analysis are the most closely related (top hits) to AVP on GENBANK at the protein level of the terminase large subunits and capsid proteins. As seen from Figure 5, according to the NCBI taxonomy database, the green-labeled leaves belong to the *Spounavirinae* subfamily. Other phages are not classified. At the level of the terminase large subunits, AVP and *Staphylococcus* phages are sister clade. On the other hand, AVP and *Bacillus* phages are sister clade in the phylogenetic tree of capsid. However, both phylogenetic trees indicated that the branch length of AVP is by far the longest branch length, suggesting AVP is phylogentically distant from all currently sequenced phages, such as *Bacillus* phages, *Listeria* phages, *Staphylococcus* phages and *Enterococcus* phages. The results indicate that AVP is a novel phage.

Six putative substructural proteins of the contractile tail of AVP were analyzed, of which the ORF81–ORF88 gene products are similar to the tail proteins of other phages. ORF81 encodes the putative tail sheath protein, which shows similarity to the sheath proteins from the published sequences of the *Enterococcus* phages EFP01 (APZ82006.1), EfV12-phi1 (AYJ73482.1), phiM1EF22 (BBE37297.1), phiEF24C (YP_001504132.1) [60], and ECP3 (YP_009147092.1). The sheath proteins are conserved in the sequence of many notoriously variable phage tail proteins. The protein encoded by ORF88 has 27–28% identity similarity to the tail fiber of these five *Enterococcus* phages.

In summary, we isolated and described the first phage that can infect *A. viridans*. The genome sequence, ORF alignment analysis, and phylogenetic tree analysis showed that AVP has extremely low similarity to other phages at the whole-genome nucleotide sequence level and at the amino acid sequence level. The observed characteristics of AVP indicate that it is a novel phage that represents a new class of phages. The results of this study have increased our understanding of phages and their diversity.

## Nucleotide Sequence Accession Numbers

The accession number of the 16S sequence of *A. viridans* AV-X1 is MK281367 in the GenBank database. The accession number of the phage AVP is MH729379 in the GenBank database. The accession number of the raw fastq files is SRR8294366 in the GenBank database.

## Supplementary Materials

The following are available online at http://www.mdpi.com/1999-4915/11/2/104/s1.
