# Supplementary material for: The Characteristics and Genome Analysis of vB_AviM_AVP, the First Phage Infecting Aerococcus viridans
Source: Viruses. 2019 Jan 26;11(2):104. doi: 10.3390/v11020104 (PMC6409932; doi:10.3390/v11020104)
Supplement: Supplementary file 1 [file viruses-11-00104-s001.zip › Table S3(Edited2).docx]

**Table S3.** General features of the putative ORFs with the best matches in the database.

| **ORF^a^** | **bp** | | **Amino Acids** | **Protein Size (kDa)** | **pI** | **Annotated Function** |
| --- | --- | --- | --- | --- | --- | --- |
|  | **Start** | **Stop** |  |  |  |  |
| 1^†^ + | 212 | 511 | 99 | 11.621 | 4.04 | No significant similarity found |
| 2^†^+ | 626 | 1000 | 124 | 14.476 | 4.28 | [Hypothetical protein](https://blast.ncbi.nlm.nih.gov/Blast.cgi" \l "alnHdr_658310449" \o "Go to alignment for hypothetical protein PHAGE6E_135 [Staphylococcus phage 6ec] >gb\|AIA64161.1\| hypothetical protein PHAGE6E_135 [Staphylococcus phage 6ec]) |
| 3^†^ + | 1594 | 1821 | 75 | 8.521 | 4.49 | Helix-turn-helix domain ([PF12728.7](http://pfam.xfam.org/family/PF12728.7)) |
| 4^†^ + | 1898 | 2209 | 103 | 12.453 | 4.91 | Putative ankyrin repeat protein |
| 5^†^ + | 2380 | 2706 | 108 | 12.562 | 5.98 | No significant similarity found |
| 6^†^+ | 2800 | 3009 | 69 | 8.035 | 9.5 | [Hypothetical protein](https://blast.ncbi.nlm.nih.gov/Blast.cgi" \l "alnHdr_655145649" \o "Go to alignment for hypothetical protein [Paenibacillus panacisoli]) |
| 7^†^ + | 3098 | 3397 | 99 | 11.2 | 9.34 | [Hypothetical protein](https://blast.ncbi.nlm.nih.gov/Blast.cgi" \l "alnHdr_655145649" \o "Go to alignment for hypothetical protein [Paenibacillus panacisoli]) |
| 8^‡^+ | 3689 | 3973 | 94 | 10.819 | 4.79 | [Hypothetical protein](https://blast.ncbi.nlm.nih.gov/Blast.cgi" \l "alnHdr_655145649" \o "Go to alignment for hypothetical protein [Paenibacillus panacisoli]) |
| 9^†^ + | 4071 | 4598 | 172 | 20.481 | 4.77 | No significant similarity found |
| 10^†^ + | 4609 | 4896 | 95 | 10.955 | 5.66 | Hemicentin-1-like |
| 11^†^ + | 5138 | 5371 | 77 | 8.845 | 5.38 | No significant similarity found |
| 12^†^+ | 5555 | 6208 | 217 | 24.275 | 5.55 | No significant similarity found |
| 13^†^ + | 6641 | 6940 | 99 | 11.548 | 4.69 | No significant similarity found |
| 14^†^ + | 7272 | 7574 | 100 | 12.193 | 4.46 | No significant similarity found |
| 15^†^ + | 8084 | 8314 | 76 | 9.150 | 3.95 | [Hypothetical protein](https://blast.ncbi.nlm.nih.gov/Blast.cgi" \l "alnHdr_971766842" \o "Go to alignment for hypothetical protein EFLK1_ORF079 [Enterococcus phage EFLK1] >gb\|AKC05038.1\| hypothetical protein EFLK1_ORF079 [Enterococcus phage EFLK1]) |
| 16^†^ + | 8647 | 8832 | 61 | 7.439 | 4.65 | No significant similarity found |
| 17^†^ + | 9058 | 9369 | 103 | 12.109 | 4.78 | Hydrolase |
| 18^†^ - | 9913 | 10,227 | 104 | 12.085 | 5.9 | [Hypothetical protein](https://blast.ncbi.nlm.nih.gov/Blast.cgi" \l "alnHdr_971766842" \o "Go to alignment for hypothetical protein EFLK1_ORF079 [Enterococcus phage EFLK1] >gb\|AKC05038.1\| hypothetical protein EFLK1_ORF079 [Enterococcus phage EFLK1]) |
| 19^†^ - | 10,698 | 11,228 | 176 | 20.598 | 4.9 | [Hypothetical protein](https://blast.ncbi.nlm.nih.gov/Blast.cgi" \l "alnHdr_971766842" \o "Go to alignment for hypothetical protein EFLK1_ORF079 [Enterococcus phage EFLK1] >gb\|AKC05038.1\| hypothetical protein EFLK1_ORF079 [Enterococcus phage EFLK1]) |
| 20^†^ - | 11,261 | 11,749 | 162 | 19.073 | 9.02 | BAH domain ([PF01426.18](http://pfam.xfam.org/family/PF01426.18)) |
| 21^†^ - | 11,749 | 11,985 | 78 | 8.859 | 4.62 | No significant similarity found |
| 22^†^ - | 11,990 | 12,586 | 198 | 22.579 | 6.61 | Putative metallo-dependent phosphatase 1 ([PF12850.7](http://pfam.xfam.org/family/PF12850.7)) |
| 23^†^ - | 12,625 | 12,855 | 76 | 9.037 | 4.42 | [Hypothetical protein](https://blast.ncbi.nlm.nih.gov/Blast.cgi" \l "alnHdr_896609754" \o "Go to alignment for hypothetical protein [Streptococcus pseudopneumoniae]) |
| 24^†^ - | 12,858 | 13,265 | 135 | 16.2 | 5.83 | [Hypothetical protein](https://blast.ncbi.nlm.nih.gov/Blast.cgi" \l "alnHdr_896609754" \o "Go to alignment for hypothetical protein [Streptococcus pseudopneumoniae]) |
| 25^†^ - | 13,268 | 13,846 | 192 | 22.277 | 5.47 | [Hypothetical protein (](https://blast.ncbi.nlm.nih.gov/Blast.cgi" \l "alnHdr_896609754" \o "Go to alignment for hypothetical protein [Streptococcus pseudopneumoniae])[PF07852.11](http://pfam.xfam.org/family/PF07852.11)[)](https://blast.ncbi.nlm.nih.gov/Blast.cgi" \l "alnHdr_896609754" \o "Go to alignment for hypothetical protein [Streptococcus pseudopneumoniae]) |
| 26^†^ - | 13,849 | 14,058 | 69 | 7.722 | 4.76 | Outer-membrane-adhesion-like protein |
| 27^†^ - | 14,061 | 14,516 | 151 | 18.171 | 4.7 | [Hypothetical protein](https://blast.ncbi.nlm.nih.gov/Blast.cgi" \l "alnHdr_1233115564" \o "Go to alignment for hypothetical protein [Aerococcus sp. 1KP-2016] >gb\|OYQ68287.1\| hypothetical protein B9P78_00315 [Aerococcus sp. 1KP-2016]) |
| 28^†^ - | 14,519 | 14,971 | 150 | 17.507 | 4.95 | [Hypothetical protein](https://blast.ncbi.nlm.nih.gov/Blast.cgi" \l "alnHdr_1233115563" \o "Go to alignment for hypothetical protein [Aerococcus sp. 1KP-2016] >gb\|OYQ68286.1\| hypothetical protein B9P78_00310 [Aerococcus sp. 1KP-2016]) |
| 29^†^ - | 14,974 | 15,429 | 151 | 18.063 | 5.03 | [Hypothetical protein](https://blast.ncbi.nlm.nih.gov/Blast.cgi" \l "alnHdr_1233115563" \o "Go to alignment for hypothetical protein [Aerococcus sp. 1KP-2016] >gb\|OYQ68286.1\| hypothetical protein B9P78_00310 [Aerococcus sp. 1KP-2016]) |
| 30^†^ - | 15,538 | 15,921 | 127 | 14.127 | 5.42 | No significant similarity found |
| 31^†^ - | 15,921 | 16,181 | 86 | 10.303 | 4.72 | [Hypothetical protein](https://blast.ncbi.nlm.nih.gov/Blast.cgi" \l "alnHdr_896219142" \o "Go to alignment for hypothetical protein [Enterococcus avium]) |
| 32^†^ - | 16,184 | 16,465 | 93 | 10.547 | 8.67 | [Hypothetical protein](https://blast.ncbi.nlm.nih.gov/Blast.cgi" \l "alnHdr_896219142" \o "Go to alignment for hypothetical protein [Enterococcus avium]) |
| 33^†^ - | 16,470 | 16,886 | 138 | 16.382 | 9.26 | No significant similarity found |
| 34^†^ - | 17,208 | 17,549 | 113 | 13.421 | 7.75 | SRP72 RNA-binding domain ([PF08492.12](http://pfam.xfam.org/family/PF08492.12)) |
| 35^†^ - | 17,610 | 18,188 | 192 | 21.825 | 4.46 | No significant similarity found |
| 36^†^ - | 18,281 | 19,462 | 393 | 45.857 | 4.74 | [RIIB protein](https://blast.ncbi.nlm.nih.gov/Blast.cgi" \l "alnHdr_414086914" \o "Go to alignment for rIIB protein [Cronobacter phage vB_CsaM_GAP31] >gb\|AFC21447.1\| rIIB protein [Cronobacter phage vB_CsaM_GAP31]) |
| 37^†^ - | 19,466 | 21,952 | 828 | 95.268 | 5.33 | [RIIA-like protein](https://blast.ncbi.nlm.nih.gov/Blast.cgi" \l "alnHdr_1227560921" \o "Go to alignment for rIIA-like protein [Streptomyces phage NootNoot] >gb\|ASR77639.1\| rIIA-like protein [Streptomyces phage Paradiddles]) |
| 38^†^ - | 22,024 | 22,275 | 83 | 9.610 | 4.63 | No significant similarity found |
| 39^†^ - | 22,279 | 22,527 | 82 | 9.437 | 5.28 | Glutaredoxin ([PF00462.24](http://pfam.xfam.org/family/PF00462.24)) |
| 40^†^ - | 22,561 | 22,755 | 64 | 7.663 | 6.55 | No significant similarity found |
| 41^†^ - | 22,765 | 22,953 | 62 | 7.263 | 4.69 | Anhydro-N-acetylmuramic acid kinase ([PF03702.14](http://pfam.xfam.org/family/PF03702.14)) |
| 42^†^ - | 23,024 | 23,455 | 143 | 16.571 | 4.59 | PGDYG protein ([PF14083.6](http://pfam.xfam.org/family/PF14083.6)) |
| 43§ - | 23,465 | 23,707 | 80 | 9.565 | 4.66 | No significant similarity found |
| 44^†^ - | 23,807 | 24,628 | 273 | 30.324 | 9.75 | [Nicotinamide mononucleotide transporter PnuC](https://blast.ncbi.nlm.nih.gov/Blast.cgi" \l "alnHdr_737465361" \o "Go to alignment for nicotinamide mononucleotide transporter PnuC [Atopococcus tabaci]) |
| 45^†^ - | 24,663 | 24,881 | 72 | 8.779 | 7.62 | Sigma factor regulator N-terminal ([PF13800.6](http://pfam.xfam.org/family/PF13800.6)) |
| 46^†^ - | 25,011 | 25,514 | 167 | 18.956 | 4.78 | Nucleoside 2-deoxyribosyltransferase ([PF05014.15](http://pfam.xfam.org/family/PF05014.15)) |
| 47^†^ - | 25,553 | 26,203 | 216 | 25.158 | 5.54 | RNA ligase ([PF09414.10](http://pfam.xfam.org/family/PF09414.10)) |
| 48^†^ - | 26,222 | 26,875 | 217 | 24.927 | 5.42 | HD domain ([PF01966.22](http://pfam.xfam.org/family/PF01966.22)) |
| 49^†^ - | 26,919 | 27,560 | 213 | 25.407 | 4.6 | Deoxyguanosine kinase ([PF01712.19](http://pfam.xfam.org/family/PF01712.19)) |
| 50^†^ - | 27,658 | 27,852 | 64 | 7.462 | 6.03 | [Hypothetical protein](https://blast.ncbi.nlm.nih.gov/Blast.cgi" \l "alnHdr_1125228819" \o "Go to alignment for hypothetical protein AUI16_06905 [Alphaproteobacteria bacterium 13_2_20CM_2_64_7]) ([PF13863.6](http://pfam.xfam.org/family/PF13863.6)) |
| 51^†^ - | 27,840 | 27,980 | 46 | 5.181 | 4.46 | [Hypothetical protein](https://blast.ncbi.nlm.nih.gov/Blast.cgi" \l "alnHdr_497310062" \o "Go to alignment for hypothetical protein [Desulfosporosinus sp. OT] >gb\|EGW36476.1\| hypothetical protein DOT_5640 [Desulfosporosinus sp. OT]) |
| 52^†^ - | 28,032 | 28,193 | 53 | 5.981 | 5.0 | [Hypothetical protein](https://blast.ncbi.nlm.nih.gov/Blast.cgi" \l "alnHdr_497310062" \o "Go to alignment for hypothetical protein [Desulfosporosinus sp. OT] >gb\|EGW36476.1\| hypothetical protein DOT_5640 [Desulfosporosinus sp. OT]) |
| 53^†^ - | 28,204 | 28,413 | 69 | 7.902 | 5.45 | [Hypothetical protein](https://blast.ncbi.nlm.nih.gov/Blast.cgi" \l "alnHdr_497310062" \o "Go to alignment for hypothetical protein [Desulfosporosinus sp. OT] >gb\|EGW36476.1\| hypothetical protein DOT_5640 [Desulfosporosinus sp. OT]) |
| 54^‡^ - | 28,526 | 28,681 | 51 | 5.788 | 5.38 | No significant similarity found |
| 55^†^ - | 28,686 | 29,169 | 161 | 18.781 | 5.02 | [Hypothetical protein](https://blast.ncbi.nlm.nih.gov/Blast.cgi" \l "alnHdr_497310062" \o "Go to alignment for hypothetical protein [Desulfosporosinus sp. OT] >gb\|EGW36476.1\| hypothetical protein DOT_5640 [Desulfosporosinus sp. OT]) |
| 56^†^ - | 29,173 | 29,433 | 86 | 10.652 | 5.5 | [Hypothetical protein (](https://blast.ncbi.nlm.nih.gov/Blast.cgi" \l "alnHdr_971765218" \o "Go to alignment for hypothetical protein EFDG1_114 [Enterococcus phage EFDG1] >gb\|AJP61420.1\| hypothetical protein EFDG1_114 [Enterococcus phage EFDG1])[PF11753.8](http://pfam.xfam.org/family/PF11753.8)[)](https://blast.ncbi.nlm.nih.gov/Blast.cgi" \l "alnHdr_971765218" \o "Go to alignment for hypothetical protein EFDG1_114 [Enterococcus phage EFDG1] >gb\|AJP61420.1\| hypothetical protein EFDG1_114 [Enterococcus phage EFDG1]) |
| 57^‡^ - | 29,426 | 29,899 | 157 | 17.978 | 4.88 | Flavivirus capsid protein C ([PF01003.19](http://pfam.xfam.org/family/PF01003.19)) |
| 58^†^- | 29,892 | 30,371 | 159 | 18.297 | 6.19 | [RNase H](https://blast.ncbi.nlm.nih.gov/Blast.cgi" \l "alnHdr_1079300525" \o "Go to alignment for ribonuclease HI [Clostridium acetireducens] >gb\|OFI00002.1\| ribonuclease HI [Clostridium acetireducens DSM 10703]) ([PF00075.24](http://pfam.xfam.org/family/PF00075.24)) |
| 59^†^ - | 30,368 | 31,006 | 212 | 23.734 | 6.08 | Thymidine kinase ([PF00265.18](http://pfam.xfam.org/family/PF00265.18)) |
| 60^†^ - | 31,176 | 31,853 | 225 | 24.854 | 4.45 | LysM domain ([PF01476.20](http://pfam.xfam.org/family/PF01476.20)) |
| 61^†^- | 31,998 | 32,816 | 272 | 31.617 | 4.81 | [Hypothetical protein](https://blast.ncbi.nlm.nih.gov/Blast.cgi" \l "alnHdr_971765218" \o "Go to alignment for hypothetical protein EFDG1_114 [Enterococcus phage EFDG1] >gb\|AJP61420.1\| hypothetical protein EFDG1_114 [Enterococcus phage EFDG1]) |
| 62^†^- | 32,833 | 33,696 | 287 | 32.682 | 4.65 | [Hypothetical protein](https://blast.ncbi.nlm.nih.gov/Blast.cgi" \l "alnHdr_971765217" \o "Go to alignment for hypothetical protein EFDG1_113 [Enterococcus phage EFDG1] >gb\|AJP61419.1\| hypothetical protein EFDG1_113 [Enterococcus phage EFDG1]) |
| 63^‡^ - | 33,755 | 34,972 | 405 | 46.528 | 5.67 | Phage terminase large subunit ([PF05876.12](http://pfam.xfam.org/family/PF05876.12)) |
| 64^†^ - | 35,092 | 36,015 | 307 | 35.262 | 9.3 | [Hypothetical protein](https://blast.ncbi.nlm.nih.gov/Blast.cgi" \l "alnHdr_849252966" \o "Go to alignment for hypothetical protein BCP8-2_036 [Bacillus phage BCP8-2] >gb\|AHJ87074.1\| hypothetical protein BCP8-2_036 [Bacillus phage BCP8-2]) |
| 65^†^ - | 36,293 | 36,838 | 181 | 20.552 | 9.93 | Phage terminase large subunit ([PF05876.12](http://pfam.xfam.org/family/PF05876.12)) |
| 66§ - | 36,831 | 37,274 | 147 | 16.148 | 4.44 | [Hypothetical protein](https://blast.ncbi.nlm.nih.gov/Blast.cgi" \l "alnHdr_971766967" \o "Go to alignment for hypothetical protein EFLK1_ORF203 [Enterococcus phage EFLK1] >gb\|AKC05163.1\| hypothetical protein EFLK1_ORF203 [Enterococcus phage EFLK1]) |
| 67§ - | 37,274 | 37,567 | 97 | 11.244 | 9.59 | [Hypothetical protein](https://blast.ncbi.nlm.nih.gov/Blast.cgi" \l "alnHdr_56693166" \o "Go to alignment for hypothetical protein LP65_gp118 [Lactobacillus virus LP65] >gb\|AAV35938.1\| orf118 [Lactobacillus virus LP65]) |
| 68^‡^+ | 37,746 | 38,225 | 159 | 18.442 | 4.91 | Intraflagellar transport complex ([PF12317.8](http://pfam.xfam.org/family/PF12317.8)) |
| 69^†^ + | 38,256 | 38,726 | 156 | 17.717 | 5.54 | Nucleotide-modification-associated domain 1 ([PF07659.11](http://pfam.xfam.org/family/PF07659.11)) |
| 70^†^ + | 39,378 | 39,704 | 108 | 12.627 | 4.64 | [Hypothetical protein](https://blast.ncbi.nlm.nih.gov/Blast.cgi" \l "alnHdr_1043846233" \o "Go to alignment for hypothetical protein vB_SscM-1_089 [Staphylococcus phage vB_SscM-1] >gb\|ANT44955.1\| hypothetical protein vB_SscM-2_088 [Staphylococcus phage vB_SscM-2]) |
| 71^†^ + | 39,733 | 41,523 | 596 | 66.863 | 5.68 | Phage portal protein ([PF04860.12](http://pfam.xfam.org/family/PF04860.12)) |
| 72^‡^ + | 41,566 | 42,372 | 268 | 30.156 | 5.08 | [Putative prohead protease](https://blast.ncbi.nlm.nih.gov/Blast.cgi" \l "alnHdr_670140492" \o "Go to alignment for putative prohead protease [Listeria phage LMTA-34]) ([PF04586.17](http://pfam.xfam.org/family/PF04586.17)) |
| 73^‡^+ | 42,377 | 43,471 | 364 | 41.894 | 4.3 | No significant similarity found |
| 74^†^ + | 43,657 | 45,126 | 489 | 53.223 | 5.13 | Major capsid protein |
| 75^†^ + | 45,218 | 45,508 | 96 | 10.979 | 9.61 | [Hypothetical protein](https://blast.ncbi.nlm.nih.gov/Blast.cgi" \l "alnHdr_1043846233" \o "Go to alignment for hypothetical protein vB_SscM-1_089 [Staphylococcus phage vB_SscM-1] >gb\|ANT44955.1\| hypothetical protein vB_SscM-2_088 [Staphylococcus phage vB_SscM-2]) |
| 76^†^ + | 45,525 | 46,475 | 316 | 34.269 | 5.43 | [Hypothetical protein](https://blast.ncbi.nlm.nih.gov/Blast.cgi" \l "alnHdr_158079314" \o "Go to alignment for hypothetical protein EFP_gp018 [Enterococcus phage phiEF24C] >ref\|YP_009147097.1\| hypothetical protein [Enterococcus phage ECP3] >ref\|YP_009219876.1\| hypothetical protein EFLK1_ORF185 [Enterococcus phage EFLK1] >dbj\|BAF81286.1\| hypothetical) |
| 77^†^ + | 46,487 | 47,338 | 283 | 32.134 | 5.36 | [Hypothetical protein](https://blast.ncbi.nlm.nih.gov/Blast.cgi" \l "alnHdr_849120022" \o "Go to alignment for hypothetical protein [Enterococcus phage ECP3] >ref\|YP_009219875.1\| hypothetical protein EFLK1_ORF184 [Enterococcus phage EFLK1] >gb\|AII28455.1\| hypothetical protein [Enterococcus phage ECP3] >gb\|AKC05144.1\| hypothetical protein EFLK1_ORF18) |
| 78^†^ + | 47,338 | 47,946 | 202 | 23.306 | 10.39 | [Hypothetical protein](https://blast.ncbi.nlm.nih.gov/Blast.cgi" \l "alnHdr_472437665" \o "Go to alignment for hypothetical protein [Bacillus phage vB_BceM_Bc431v3] >gb\|AFQ96536.1\| hypothetical protein [Bacillus phage vB_BceM_Bc431v3]) |
| 79^†^ + | 47,992 | 48,819 | 275 | 31.295 | 4.95 | [Hypothetical protein](https://blast.ncbi.nlm.nih.gov/Blast.cgi" \l "alnHdr_971765234" \o "Go to alignment for hypothetical protein EFDG1_130 [Enterococcus phage EFDG1] >gb\|AJP61436.1\| hypothetical protein EFDG1_130 [Enterococcus phage EFDG1]) |
| 80^‡^ + | 48,833 | 49,108 | 91 | 10.457 | 9.59 | [Hypothetical protein](https://blast.ncbi.nlm.nih.gov/Blast.cgi" \l "alnHdr_1139676374" \o "Go to alignment for hypothetical protein EFP01_080 [Enterococcus phage EFP01]) |
| 81^†^ + | 49,114 | 50,820 | 568 | 61.255 | 4.85 | Phage tail sheath protein ([PF17482.2](http://pfam.xfam.org/family/PF17482.2)) |
| 82^†^ + | 50,840 | 51,268 | 142 | 15.574 | 5.55 | [Tail tube subunit](https://blast.ncbi.nlm.nih.gov/Blast.cgi" \l "alnHdr_971765237" \o "Go to alignment for tail tube subunit [Enterococcus phage EFDG1] >gb\|AJP61439.1\| tail tube subunit [Enterococcus phage EFDG1] >gb\|APZ82005.1\| tail tube subunit [Enterococcus phage EFP01]) |
| 83^†^ + | 51,384 | 51,821 | 145 | 16.81 | 5.03 | Tail assembly chaperone |
| 84^†^ + | 51,886 | 52,413 | 175 | 20.806 | 4.46 | Putative RNA polymerase |
| 85^‡^ + | 52,430 | 56,293 | 1287 | 136.86 | 5.36 | Mannosyl-glycoproten endo-beta-N-acetylglucosaminidase ([PF01832.20](http://pfam.xfam.org/family/PF01832.20)) |
| 86^†^ + | 56,393 | 58,369 | 658 | 73.708 | 4.84 | Tail lysin-like protein ([PF01551.22](http://pfam.xfam.org/family/PF01551.22)) |
| 87^†^ + | 58,436 | 58,879 | 147 | 16.34 | 6.83 | [Putative tail lysin](https://blast.ncbi.nlm.nih.gov/Blast.cgi" \l "alnHdr_656632702" \o "Go to alignment for putative tail lysin [Listeria phage LMTA-94]) |
| 88§ + | 58,893 | 62,849 | 1318 | 148.617 | 4.89 | [Putative tail fiber](https://blast.ncbi.nlm.nih.gov/Blast.cgi" \l "alnHdr_971765242" \o "Go to alignment for putative tail fiber [Enterococcus phage EFDG1] >gb\|AJP61444.1\| putative tail fiber [Enterococcus phage EFDG1]) |
| 89^‡^ + | 62,846 | 67,510 | 1554 | 167.614 | 5.29 | Putative anti-receptor protein |
| 90^†^ + | 67,586 | 70,624 | 1012 | 112.082 | 5.36 | Receptor-binding protein |
| 91^‡^ + | 70,640 | 72,724 | 694 | 73.821 | 9.27 | [Hypothetical protein](https://blast.ncbi.nlm.nih.gov/Blast.cgi" \l "alnHdr_1227033066" \o "Go to alignment for hypothetical protein [Bacillus licheniformis]) [(](https://blast.ncbi.nlm.nih.gov/Blast.cgi" \l "alnHdr_748651239" \o "Go to alignment for pilus assembly protein [[Clostridium] scindens])[PF05895.12](http://pfam.xfam.org/family/PF05895.12)[)](https://blast.ncbi.nlm.nih.gov/Blast.cgi" \l "alnHdr_748651239" \o "Go to alignment for pilus assembly protein [[Clostridium] scindens]) |
| 92^†^ + | 72,737 | 73,717 | 326 | 37.56 | 3.98 | No significant similarity found |
| 93^†^ + | 73,710 | 73,874 | 54 | 6.579 | 5.29 | No significant similarity found |
| 94^†^ + | 73,876 | 74,904 | 342 | 38.217 | 4.55 | Hypothetical protein |
| 95^‡^ + | 74,990 | 75,454 | 154 | 17.156 | 4.46 | Bacteriophage holin ([PF04531.13](http://pfam.xfam.org/family/PF04531.13)) |
| 96^†^ + | 75,568 | 77,055 | 495 | 55.369 | 9.29 | LysM domain ([PF01476.20](http://pfam.xfam.org/family/PF01476.20)) |
| 97^†^ + | 77,206 | 77,409 | 67 | 7.590 | 9.49 | No significant similarity found |
| 98^†^ + | 77,409 | 77,945 | 178 | 20.36 | 4.9 | LysM peptidoglycan-binding domain-containing protein |
| 99^†^ + | 78,059 | 78,658 | 199 | 23.25 | 5.74 | [Hypothetical protein](https://blast.ncbi.nlm.nih.gov/Blast.cgi" \l "alnHdr_589890817" \o "Go to alignment for hypothetical protein T548_0058 [Lactococcus phage phiL47] >gb\|AHC94136.1\| hypothetical protein T548_0058 [Lactococcus phage phiL47]) |
| 100^‡^ + | 78,670 | 79,359 | 229 | 24.642 | 4.81 | Cell wall hydrolase ([PF07486.12](http://pfam.xfam.org/family/PF07486.12)) |
| 101^†^ + | 79,443 | 79,940 | 165 | 19.683 | 5.1 | [Hypothetical protein](https://blast.ncbi.nlm.nih.gov/Blast.cgi" \l "alnHdr_1008239398" \o "Go to alignment for hypothetical protein [Streptococcus oralis] >gb\|KXU16391.1\| hypothetical protein SORDD17_00400 [Streptococcus oralis]) |
| 102^†^ + | 79,940 | 80,164 | 74 | 8.760 | 8.76 | No significant similarity found |
| 103^†^ + | 80,301 | 80,666 | 121 | 14.06 | 5.72 | Putative ankyrin-repeat protein |
| 104^†^ + | 80,732 | 81,490 | 252 | 27.588 | 5.97 | Gp31 |
| 105^†^ + | 81,490 | 82,011 | 173 | 20.196 | 8.47 | [Hypothetical protein](https://blast.ncbi.nlm.nih.gov/Blast.cgi" \l "alnHdr_158079332" \o "Go to alignment for hypothetical protein EFP_gp036 [Enterococcus phage phiEF24C] >dbj\|BAF81304.1\| hypothetical protein EFP_036 [Enterococcus phage phiEF24C]) |
| 106^†^ + | 81,998 | 82,699 | 233 | 26.591 | 5.25 | Baseplate protein |
| 107^†^ + | 82,710 | 83,759 | 349 | 39.518 | 5.0 | Baseplate_J﻿ ([PF04865.14](http://pfam.xfam.org/family/PF04865.14)) |
| 108^†^ + | 83,776 | 85,938 | 720 | 82.805 | 5.36 | [Hypothetical protein](https://blast.ncbi.nlm.nih.gov/Blast.cgi" \l "alnHdr_158079335" \o "Go to alignment for hypothetical protein EFP_gp039 [Enterococcus phage phiEF24C] >dbj\|BAF81307.1\| hypothetical protein EFP_039 [Enterococcus phage phiEF24C]) |
| 109^‡^ + | 85,980 | 86,501 | 173 | 19.484 | 9.32 | Baseplate protein |
| 110^‡^ + | 86,523 | 90,002 | 1159 | 128.451 | 5.28 | Putative tail protein |
| 111^†^ + | 90,161 | 90,409 | 82 | 9.174 | 5.31 | [Hypothetical protein](https://blast.ncbi.nlm.nih.gov/Blast.cgi" \l "alnHdr_658307601" \o "Go to alignment for hypothetical protein [Listeria phage List-36] >ref\|YP_009042914.1\| hypothetical protein LP048_106 [Listeria phage LP-048] >ref\|YP_009055702.1\| hypothetical protein [Listeria phage LMTA-148] >gb\|AHL19779.1\| hypothetical protein LP048_106 [Li) |
| 112^†^ + | 90,443 | 90,607 | 54 | 6.213 | 4.21 | [Hypothetical protein](https://blast.ncbi.nlm.nih.gov/Blast.cgi" \l "alnHdr_1056548145" \o "Go to alignment for hypothetical protein [Aerococcus urinaehominis] >gb\|AMB99902.1\| hypothetical protein AWM75_07935 [Aerococcus urinaehominis] >emb\|SDM52428.1\| hypothetical protein SAMN04487985_12111 [Aerococcus urinaehominis]) |
| 113^†^ - | 92,778 | 93,161 | 127 | 14.256 | 5.61 | [Hypothetical protein](https://blast.ncbi.nlm.nih.gov/Blast.cgi" \l "alnHdr_1056548145" \o "Go to alignment for hypothetical protein [Aerococcus urinaehominis] >gb\|AMB99902.1\| hypothetical protein AWM75_07935 [Aerococcus urinaehominis] >emb\|SDM52428.1\| hypothetical protein SAMN04487985_12111 [Aerococcus urinaehominis]) |
| 114^†^ + | 94,425 | 96,479 | 684 | 77.702 | 5.74 | DNA helicase ([PF00271.31](http://pfam.xfam.org/family/PF00271.31)) |
| 115^‡^+ | 96,514 | 98,322 | 602 | 70.752 | 5.78 | Putative transcriptional regulator |
| 116^†^ + | 98,390 | 99,853 | 487 | 54.8 | 5.42 | Helicase |
| 117^†^ + | 99,853 | 100,881 | 342 | 38.687 | 5.46 | [Recombination exonuclease](https://blast.ncbi.nlm.nih.gov/Blast.cgi" \l "alnHdr_670140461" \o "Go to alignment for recombination exonuclease [Listeria phage LMTA-34]) |
| 118^†^ + | 100,971 | 102,929 | 652 | 74.735 | 5.25 | [Putative exonuclease](https://blast.ncbi.nlm.nih.gov/Blast.cgi" \l "alnHdr_658607567" \o "Go to alignment for putative exonuclease [Listeria phage LP-083-2] >gb\|AHL19336.1\| putative exonuclease [Listeria phage LP-083-2] >gb\|AHL19447.1\| putative exonuclease [Listeria phage LP-124]) |
| 119^†^ + | 102,933 | 103,373 | 146 | 17.433 | 5.0 | No significant similarity found |
| 120^†^ + | 103,384 | 103,665 | 93 | 10.929 | 4.66 | No significant similarity found |
| 121^†^ + | 103,708 | 104,760 | 350 | 40.343 | 5.98 | [Putative](https://blast.ncbi.nlm.nih.gov/Blast.cgi" \l "alnHdr_1139676347" \o "Go to alignment for putative primase [Enterococcus phage EFP01]) [primase](https://blast.ncbi.nlm.nih.gov/Blast.cgi" \l "alnHdr_1139676347" \o "Go to alignment for putative primase [Enterococcus phage EFP01]) |
| 122^†^ + | 104,772 | 105,464 | 230 | 26.81 | 4.31 | No significant similarity found |
| 123^†^ + | 105,457 | 105,783 | 108 | 12.516 | 4.13 | No significant similarity found |
| 124^†^ + | 105,770 | 106,213 | 147 | 16.902 | 5.08 | [Hypothetical protein](https://blast.ncbi.nlm.nih.gov/Blast.cgi" \l "alnHdr_1056548145" \o "Go to alignment for hypothetical protein [Aerococcus urinaehominis] >gb\|AMB99902.1\| hypothetical protein AWM75_07935 [Aerococcus urinaehominis] >emb\|SDM52428.1\| hypothetical protein SAMN04487985_12111 [Aerococcus urinaehominis]) |
| 125^‡^+ | 106,214 | 106,849 | 211 | 24.248 | 5.5 | Resolvase |
| 126^‡^+ | 106,861 | 107,580 | 239 | 28.87 | 7.54 | [Hypothetical protein](https://blast.ncbi.nlm.nih.gov/Blast.cgi" \l "alnHdr_1056548145" \o "Go to alignment for hypothetical protein [Aerococcus urinaehominis] >gb\|AMB99902.1\| hypothetical protein AWM75_07935 [Aerococcus urinaehominis] >emb\|SDM52428.1\| hypothetical protein SAMN04487985_12111 [Aerococcus urinaehominis]) |
| 127^†^ + | 107,586 | 107,930 | 114 | 12.917 | 8.67 | Integration host factor |
| 128^‡^+ | 108,057 | 110,297 | 746 | 86.404 | 5.49 | DNA polymerase ([PF00476.20](http://pfam.xfam.org/family/PF00476.20)) |
| 129^†^ + | 110,453 | 111,148 | 231 | 27.203 | 9.24 | HNH homing endonuclease 1 |
| 130^‡^+ | 111,323 | 112,024 | 233 | 26.789 | 5.58 | DNA polymerase ([PF00476.20](http://pfam.xfam.org/family/PF00476.20)) |
| 131^†^ + | 112,029 | 112,214 | 61 | 7.171 | 8.73 | Transcription factor |
| 132^†^ + | 112,214 | 11,2696 | 160 | 18.864 | 5.21 | [Hypothetical protein](https://blast.ncbi.nlm.nih.gov/Blast.cgi" \l "alnHdr_158079358" \o "Go to alignment for hypothetical protein EFP_gp062 [Enterococcus phage phiEF24C] >ref\|YP_009147046.1\| hypothetical protein [Enterococcus phage ECP3] >ref\|YP_009219829.1\| hypothetical protein EFLK1_ORF138 [Enterococcus phage EFLK1] >dbj\|BAF81330.1\| hypothetical) |
| 133^†^ + | 112,844 | 114,013 | 389 | 42.758 | 4.74 | [Hypothetical protein](https://blast.ncbi.nlm.nih.gov/Blast.cgi" \l "alnHdr_1139676325" \o "Go to alignment for hypothetical protein EFP01_031 [Enterococcus phage EFP01]) |
| 134§+ | 114,066 | 114,344 | 92 | 11.171 | 9.14 | No significant similarity found |
| 135^‡^+ | 114,357 | 115,595 | 412 | 46.024 | 5.0 | Putative recombinase A ([PF00154.21](http://pfam.xfam.org/family/PF00154.21)) |
| 136^†^ + | 115,582 | 115,923 | 113 | 13.052 | 5.23 | [Hypothetical protein](https://blast.ncbi.nlm.nih.gov/Blast.cgi" \l "alnHdr_1043846293" \o "Go to alignment for hypothetical protein vB_SscM-1_149 [Staphylococcus phage vB_SscM-1] >gb\|ANT45015.1\| hypothetical protein vB_SscM-2_148 [Staphylococcus phage vB_SscM-2]) |
| 137^†^ + | 115,916 | 116,545 | 209 | 25.269 | 7.54 | Gp143 |
| 138^†^ + | 116,653 | 117,222 | 189 | 20.761 | 4.55 | Ig-like domain-containing protein |
| 139^†^ + | 117,565 | 118,467 | 300 | 34.457 | 5.01 | No significant similarity found |
| 140^‡^+ | 118,481 | 119,746 | 421 | 48.367 | 5.18 | Mre11 nuclease |
| 141^†^ + | 119,750 | 120,217 | 155 | 18.192 | 7.25 | No significant similarity found |
| 142^†^ + | 120,204 | 120,851 | 215 | 24.518 | 5.26 | Gp82 |
| 143^†^ + | 120,902 | 121,072 | 56 | 6.600 | 6.34 | [Hypothetical protein](https://blast.ncbi.nlm.nih.gov/Blast.cgi" \l "alnHdr_658307460" \o "Go to alignment for hypothetical protein [Listeria phage LMSP-25] >gb\|AIA64516.1\| hypothetical protein [Listeria phage LMSP-25] >gb\|AID17074.1\| hypothetical protein [Listeria phage LMTA-34]) |
| 144^†^ + | 121,075 | 121,254 | 59 | 7.424 | 9.81 | No significant similarity found |
| 145^‡^+ | 121,251 | 12,2021 | 256 | 29.59 | 8.91 | Gp152 |
| 146^†^ + | 122,014 | 122,400 | 128 | 14.828 | 9.55 | [Hypothetical protein](https://blast.ncbi.nlm.nih.gov/Blast.cgi" \l "alnHdr_643217619" \o "Go to alignment for hypothetical protein [Bacillus phage Evoli] >gb\|AHZ09924.1\| hypothetical protein [Bacillus phage Evoli]) |
| 147§+ | 122,400 | 123,281 | 293 | 33.668 | 5.27 | [Hypothetical protein](https://blast.ncbi.nlm.nih.gov/Blast.cgi" \l "alnHdr_472437852" \o "Go to alignment for hypothetical protein Romulus_095 [Staphylococcus phage vB_SauM_Romulus] >ref\|YP_008431214.1\| hypothetical protein Remus_095 [Staphylococcus phage vB_SauM_Remus] >gb\|AFV80974.1\| hypothetical protein Remus_095 [Staphylococcus phage vB_SauM_Re) |
| 148^†^ + | 123,716 | 124,405 | 229 | 25.911 | 5.88 | [Hypothetical protein](https://blast.ncbi.nlm.nih.gov/Blast.cgi" \l "alnHdr_1139676304" \o "Go to alignment for hypothetical protein EFP01_010 [Enterococcus phage EFP01]) |
| 149^‡^+ | 124,463 | 124,978 | 171 | 20.128 | 9.47 | No significant similarity found |
| 150^†^ + | 124,981 | 125,382 | 133 | 15.434 | 5.81 | [Hypothetical protein](https://blast.ncbi.nlm.nih.gov/Blast.cgi" \l "alnHdr_1219322927" \o "Go to alignment for hypothetical protein [Blastococcus sp. DSM 44272]) |
| 151^†^ + | 125,386 | 125,859 | 157 | 18.634 | 4.45 | No significant similarity found |
| 152^†^ + | 125,861 | 126,058 | 65 | 7.392 | 7.67 | [Hypothetical protein](https://blast.ncbi.nlm.nih.gov/Blast.cgi" \l "alnHdr_564292650" \o "Go to alignment for hypothetical protein [Bacillus phage vB_BanS-Tsamsa] >gb\|AGI11827.1\| hypothetical protein [Bacillus phage vB_BanS-Tsamsa]) |
| 153^†^ + | 126,289 | 126,657 | 122 | 14.531 | 9.73 | [Hypothetical protein](https://blast.ncbi.nlm.nih.gov/Blast.cgi" \l "alnHdr_564292650" \o "Go to alignment for hypothetical protein [Bacillus phage vB_BanS-Tsamsa] >gb\|AGI11827.1\| hypothetical protein [Bacillus phage vB_BanS-Tsamsa]) |
| 154^†^ + | 126,669 | 127,184 | 171 | 20.456 | 4.53 | [Hypothetical protein](https://blast.ncbi.nlm.nih.gov/Blast.cgi" \l "alnHdr_984928255" \o "Go to alignment for hypothetical protein [Aerococcus urinae] >gb\|AMB95986.1\| hypothetical protein AWM73_05445 [Aerococcus urinae] >gb\|ORE69769.1\| hypothetical protein B6C83_06930 [Aerococcus urinae]) |
| 155^†^ + | 127,318 | 127,557 | 79 | 9.343 | 4.34 | [Hypothetical protein](https://blast.ncbi.nlm.nih.gov/Blast.cgi" \l "alnHdr_984928255" \o "Go to alignment for hypothetical protein [Aerococcus urinae] >gb\|AMB95986.1\| hypothetical protein AWM73_05445 [Aerococcus urinae] >gb\|ORE69769.1\| hypothetical protein B6C83_06930 [Aerococcus urinae]) |
| 156^†^ + | 127,734 | 127,874 | 46 | 5.348 | 11.11 | No significant similarity found |
| 157^†^ + | 127,895 | 128,041 | 48 | 6.0 | 10.66 | No significant similarity found |
| 158^†^ + | 128,077 | 128,445 | 122 | 14.167 | 5.58 | [Hypothetical protein](https://blast.ncbi.nlm.nih.gov/Blast.cgi" \l "alnHdr_1268562273" \o "Go to alignment for hypothetical protein [Bacillus cereus] >gb\|PGS22366.1\| hypothetical protein COC59_20430 [Bacillus cereus]) |
| 159^†^ + | 128,548 | 128,817 | 89 | 10.58 | 4.89 | No significant similarity found |
| 160^†^ + | 130,044 | 130,736 | 230 | 27.3 | 4.05 | [Hypothetical protein](https://blast.ncbi.nlm.nih.gov/Blast.cgi" \l "alnHdr_158079440" \o "Go to alignment for hypothetical protein EFP_gp144 [Enterococcus phage phiEF24C] >dbj\|BAF81412.1\| hypothetical protein EFP_144 [Enterococcus phage phiEF24C]) |
| 161^†^ + | 130,968 | 131,633 | 221 | 25.09 | 4.21 | [Hypothetical protein](https://blast.ncbi.nlm.nih.gov/Blast.cgi" \l "alnHdr_158079440" \o "Go to alignment for hypothetical protein EFP_gp144 [Enterococcus phage phiEF24C] >dbj\|BAF81412.1\| hypothetical protein EFP_144 [Enterococcus phage phiEF24C]) |
| 162^†^ + | 131,743 | 132,054 | 103 | 11.59 | 9.36 | [Hypothetical protein](https://blast.ncbi.nlm.nih.gov/Blast.cgi" \l "alnHdr_158079440" \o "Go to alignment for hypothetical protein EFP_gp144 [Enterococcus phage phiEF24C] >dbj\|BAF81412.1\| hypothetical protein EFP_144 [Enterococcus phage phiEF24C]) |
| 163^†^ + | 132,122 | 132,385 | 87 | 10.067 | 5.22 | Putative ankyrin-repeat protein |
| 164^†^ + | 132,389 | 132,739 | 116 | 14.07 | 8.53 | No significant similarity found. |
| 165^†^ + | 132,881 | 133,537 | 218 | 26.137 | 3.98 | No significant similarity found. |

^a^ ^†^ AUG start codons; ^‡^ UUG start codons; ^§^ GUG start codons. ^+^ right orientation; ^-^ left orientation.
