# Supplementary material for: The Characteristics and Genome Analysis of vB_AviM_AVP, the First Phage Infecting Aerococcus viridans
Source: Viruses. 2019 Jan 26;11(2):104. doi: 10.3390/v11020104 (PMC6409932; doi:10.3390/v11020104)
Supplement: Supplementary file 1 [file viruses-11-00104-s001.zip › Table S2(Edited2).docx]

**Table S2.** Transfer-RNA-encoding genes of vB_AviM_AVP (AVP).

| **tRNA#** | **tRNA Begin** | **tRNA End** | **tRNA Type** | **Anticodon** |
| --- | --- | --- | --- | --- |
| 1 | 93,394 | 93,323 | Arg | TCT |
| 2 | 92,368 | 92,296 | Thr | TGT |
| 3 | 92,291 | 92,204 | Ser | GCT |
| 4 | 92,108 | 92,035 | Asn | GTT |
| 5 | 92,021 | 91,939 | Tyr | GTA |
