# Supplementary material for: The Characteristics and Genome Analysis of vB_AviM_AVP, the First Phage Infecting Aerococcus viridans
Source: Viruses. 2019 Jan 26;11(2):104. doi: 10.3390/v11020104 (PMC6409932; doi:10.3390/v11020104)
Supplement: Supplementary file 1 [file viruses-11-00104-s001.zip › Supplementary files(Edited).docx]

**Supplementary files:**

**
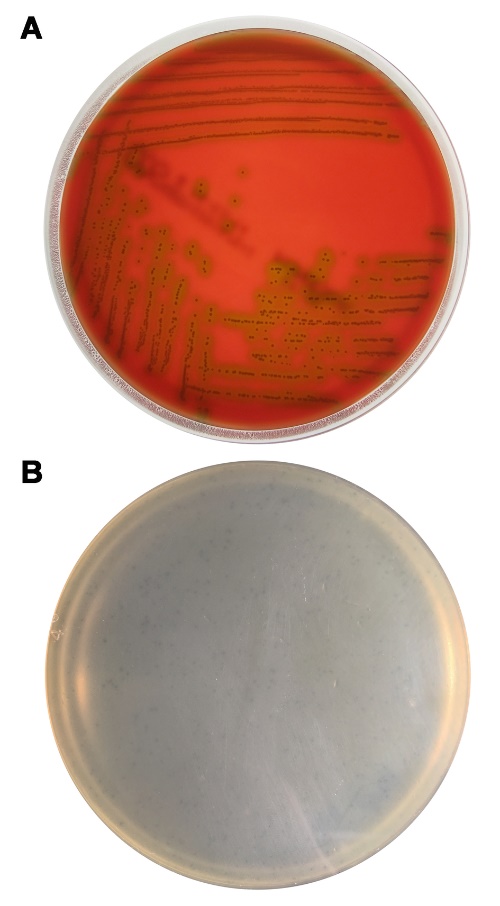
**

**Figure S1. (a)** Colony morphology. On 5% sheep-blood agar, Aerococcus viridans colonies were ≤1 mm in size, α-hemolytic, and had an off-white color. **(b)** Plaques of AVP. An AVP stock solution (0.1 mL) was mixed with an AV-X1 strain (0.2 mL) in 7 mL semisolid BHI medium (0.75% agar) and was transferred directly onto a solidified base nutrient agar (1.5% agar). After 8 h of incubation at 37 °C, the plates were assessed for phage plaques.


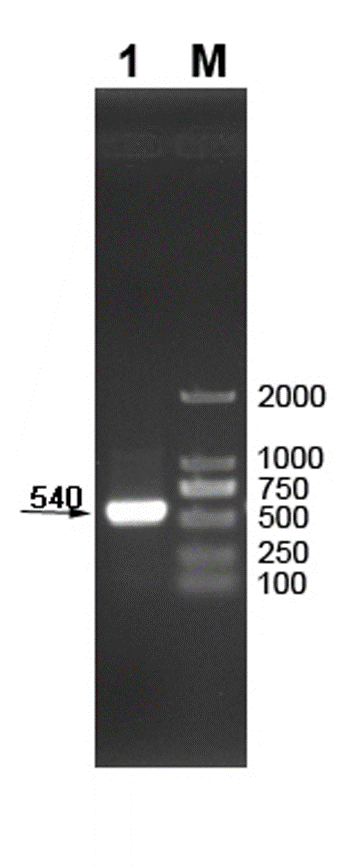


**Figure S2.** The results of PCR (16S rRNA) with *Aerococcus viridans*–specific primers. Lane 1 contains a PCR fragment of AV-X; lane M contains a 2000-bp DNA marker. The 16S sequence of *A. viridans* AV-X1 has been submitted to the NCBI and the accession number is MK281367.


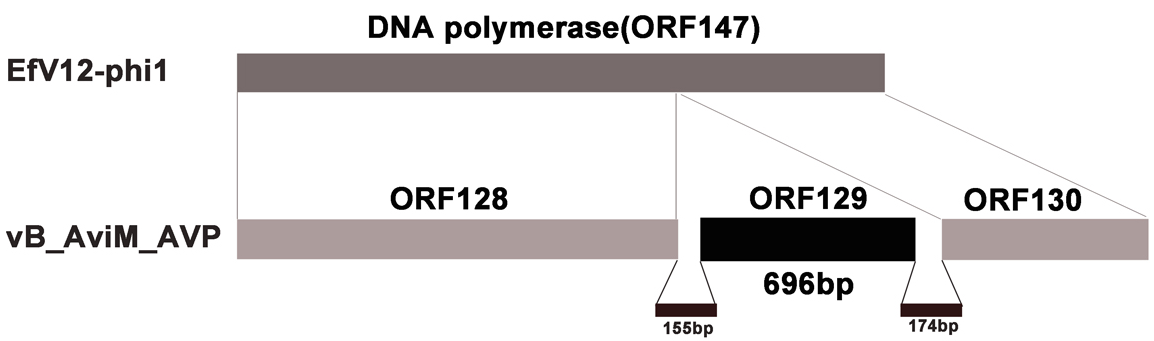


**Figure S3.** Schematic representation of genes related to the phages vB_AviM_AVP (AVP) and EfV12-phi1, with an inserted gene fragment (1025 bp), which contains the HNH endonuclease-like ORF129 (696 bp) in between two small gene fragments with sizes of 155 bp and 174 bp.
