# Supplementary material for: The Characteristics and Genome Analysis of vB_AviM_AVP, the First Phage Infecting Aerococcus viridans
Source: Viruses. 2019 Jan 26;11(2):104. doi: 10.3390/v11020104 (PMC6409932; doi:10.3390/v11020104)
Supplement: Supplementary file 1 [file viruses-11-00104-s001.zip › Table S1(Edited).docx]

**Table S1.** The biochemical tests of *Aerococcus viridans* AV-X1.

| **No.** | **Tests** | **+/-** | **No.** | **Tests** | **+/-** |
| --- | --- | --- | --- | --- | --- |
| 1 | Amygdalin (AMY) | **-** | 23 | D-mannitol (dMAN) | **+** |
| 2 | Alanine-phenylalanine-proline-aromatase (APPA) | **-** | 24 | Salicin (SAL) | **+** |
| 3 | Leucine aromatase (LeuA) | **-** | 25 | Arginine dihydrolase I (ADH1) | **-** |
| 4 | Alanine aromatase (AiaA) | **-** | 26 | β-galactosylpyranosidase (BGAR) | **-** |
| 5 | D-ribose (dRIB) | **+** | 27 | α-galactosidase (AGAL) | **-** |
| 6 | Neomycin tolerance (NOVO) | **+** | 28 | Urase (URE) | **-** |
| 7 | D-raffinose (dRAF) | **+** | 29 | N-acetyl-D-glucosamine (NAG) | **+** |
| 8 | Optoxin tolerance (OPTO) | **+** | 30 | D-mannose (dMNE) | **+** |
| 9 | Phosphatidylphospholipase-C (PIPLC) | **-** | 31 | Saccharose (SAC) | **+** |
| 10 | Cyclodextrin (CDEX) | **-** | 32 | β-D-galactosidase (BGAL) | **-** |
| 11 | L-proline aromatase (ProA) | **-** | 33 | α-mannosidase (AMAN) | **-** |
| 12 | Tyrosine aromatase (TyrA) | **-** | 34 | Pyroglutamic acid aromatase (PyrA) | **+** |
| 13 | L-lactate alkali production (ILATk) | **-** | 35 | Polymyxin B tolerance (POLYB) | **-** |
| 14 | 6.5% NaCl (NC6.5) | **+** | 36 | D-malt dust (dMAL) | **+** |
| 15 | O/129 resistance (O129R) | **+** | 37 | Methyl-B-D- glucopyranoside (MBdG) | **+** |
| 16 | D-xylose (dXYL) | **-** | 38 | D-trehalose (dTRE) | **+** |
| 17 | L-aspartate aromatase (AspA) | **-** | 39 | α-glucosaccharase (AGLU) | **-** |
| 18 | β-glucuronidase (BGURr) | **-** | 40 | Phosphatase (PHOS) | **-** |
| 19 | D-sorbierite (dSOR) | **+** | 41 | β-D-glucuronidase (BGUR) | **-** |
| 20 | Lactose (LAC) | **-** | 42 | Amylopectin (PUL) | **-** |
| 21 | D-galactose (dGAL) | **-** | 43 | Arginine dihydrolase 2 (ADH2s) | **-** |
| 22 | Bacillary peptide tolerance (BACI) | **-** |  |  |  |
